# Sterility of *Aedes albopictus* by X-ray Irradiation as an Alternative to γ-ray Irradiation for the Sterile Insect Technique

**DOI:** 10.3390/pathogens12010102

**Published:** 2023-01-06

**Authors:** Lin-Min Wang, Ni Li, Cui-Ping Ren, Zhe-Yu Peng, Hong-Zheng Lu, Dong Li, Xin-Yu Wu, Zi-Xin Zhou, Jian-Yi Deng, Zi-Han Zheng, Ruo-Qing Wang, Yi-Nan Du, Duo-Quan Wang, Sheng-Qun Deng

**Affiliations:** 1 The Key Laboratory of Microbiology and Parasitology of Anhui Province, the Key Laboratory of Zoonoses of High Institutions in Anhui, Department of Pathogen Biology, School of Basic Medical Sciences, Anhui Medical University, Hefei 230032, China; 2245010044@stu.ahmu.edu.cn (L.-M.W.); 1913020002@stu.ahmu.edu.cn (N.L.); cuipingren@126.com (C.-P.R.); pengzheyu@stu.ahmu.edu.cn (Z.-Y.P.); luhongzheng@stu.ahmu.edu.cn (H.-Z.L.); m18684752925@163.com (D.L.); zhuzongsz@163.com (X.-Y.W.); zzhouzixin@163.com (Z.-X.Z.); dengjianyi_dd@163.com (J.-Y.D.); zhengzihan0720@163.com (Z.-H.Z.); wangruoqing@stu.ahmu.edu.cn (R.-Q.W.); 2 National Institute of Parasitic Diseases, Chinese Center for Disease Control and Prevention (Chinese Center for Tropical Diseases Research), National Health Commission Key Laboratory of Parasite and Vector Biology, WHO Collaborating Center for Tropical Diseases, National Center for International Research on Tropical Diseases, Shanghai 200025, China

**Keywords:** sterile insect technology, pupal radiation, radiation dose, induced sterility, male mating competitiveness

## Abstract

The mosquito *Aedes albopictus* can transmit various arboviral diseases, posing a severe threat to human health. As an environmentally friendly method, sterile insect technology (SIT) is considered an alternative to traditional methods such as chemical pesticides to control *Ae. albopictus*. In SIT, the sterility of male mosquitoes can be achieved by γ-ray or X-ray radiation. Compared with γ-rays, X-rays are easier to obtain, cheaper, and less harmful. However, there is a lack of comparative assessment of these two types of radiation for SIT under the same controlled conditions. Here, we compared the effects of X-ray and γ-ray radiation on the sterility of *Ae. albopictus* males under laboratory-controlled conditions. Neither type of radiation affected the number of eggs but significantly reduced the survival time and hatch rate. The same dose of γ-rays caused a higher sterility effect on males than X-rays but had a more significant impact on survival. However, X-rays could achieve the same sterility effect as γ-rays by increasing the radiation dose. For example, X-rays of 60 Gy induced 99% sterility, similar to γ-rays of 40 Gy. In the test of male mating competitiveness, the induced sterility and the male mating competitiveness index were also identical at the same release ratio (sterile males/fertile males). At a release ratio of 7:1, nearly 80% of eggs failed to hatch. Sterile males produced by X-ray and γ-ray radiation had similar male competitiveness in competition with field males. In conclusion, a higher dose of X-rays is required to achieve the same sterility effect, compared to γ-rays. When γ-rays are not readily available, high-dose X-rays can be used instead. This study provides data supporting the selection of more suitable radiation for the field release of sterile male mosquitoes.

## 1. Introduction

The Asian tiger mosquito, *Aedes albopictus*, has spread from southeast Asia to all continents except Antarctica and is considered one of the world’s top 100 invasive species. [1,2,3]. It transmits various viral diseases, such as yellow fever, dengue, chikungunya, and Zika, which significantly burden human health [4,5]. Without effective arbovirus vaccines and drugs, disease control relies heavily on suppressing mosquito populations, with chemical insecticides being the most commonly used method [6,7]. However, most chemical insecticides act not only on mosquitoes but also on other nontarget organisms. Additionally, chemical insecticides pose different problems, including the emergence of resistance and human toxicity [8,9]. Therefore, developing new environmentally friendly methods to control mosquito populations is imperative.

Sterile insect technology (SIT), which is species-specific, eco-friendly, and environmentally sound, reduces mosquito populations by releasing massive numbers of sterile males into the wild to mate with females [10]. This technique has been successfully applied to control several pests of agricultural and veterinary importance [11,12]. However, for mosquito vectors of human diseases, the development of SIT is still at an early stage. Pilot trials are being conducted to determine whether SIT is an effective method for the population control of mosquito species, including *Aedes* spp. [13,14]. In some areas of Europe colonized by *Ae. albopictus* mosquitoes, SIT has achieved the goal of controlling populations without causing environmental harm [15]. In field experiments in Italy, when egg sterility reached 81%, it effectively suppressed the local mosquito population [16]. Recently, *Ae. albopictus* population has been dramatically reduced by SIT in the trial areas of Freiburg and Ludwigshafen, where the egg sterility was 62.7% and 84.7%, respectively [17].

In SIT, sterilization of male mosquitoes before release can be achieved by irradiation [18]. Gamma-ray irradiation systems are usually used for this purpose. Nevertheless, because of the fear of terrorism, the control of radioisotopes has become increasingly strict, making them increasingly difficult to purchase, transport, or reload [19,20]. Therefore, low-energy X-ray irradiation systems are a good alternative, as they present some positive characteristics such as easy accessibility, discontinuous emission of radiation, low harmfulness, simple operation, and low cost [21,22,23]. There have been many studies on using γ-ray radiation-based SIT to control *Ae. albopictus*, but relatively few studies have focused on using X-ray radiation-based SIT.

To investigate the different effects of X-rays and γ-rays in SIT for *Ae. albopictus* control, we compared the sterility effect of X-ray and γ-ray radiation on male mosquitoes. In this study, male pupae of *Ae. albopictus* were irradiated with different doses of X-rays or γ-rays. The emergence rate, survival time, number of eggs, hatch rate, induced sterility, and male mating competitiveness index were measured to compare the radiation effects of X-rays and γ-rays. This study is of great significance for using SIT to suppress *Ae. albopictus* populations.

## 2. Results

### 2.1. Effects of Male Pupae Radiation on the Emergence Rate, Egg Number, and Egg Hatch Rate

For emergence rate, the male pupae exposed to any dose of X-rays were not significantly different from the control group (χ^2^ = 5.881, df = 6, *p* = 0.437) (Table 1). However, the emergence rate was lower than that of the control group when the dose of γ-rays reached 50 and 60 Gy (χ^2^ = 66.121, df = 6, *p* < 0.001). When exposed to 60 Gy, the emergence rate of pupae was statistically lower upon γ-ray irradiation (75.6 ± 3.1%) than upon X-ray irradiation (89.6 ± 4.3%) (Table 1).

**Figure 1:**
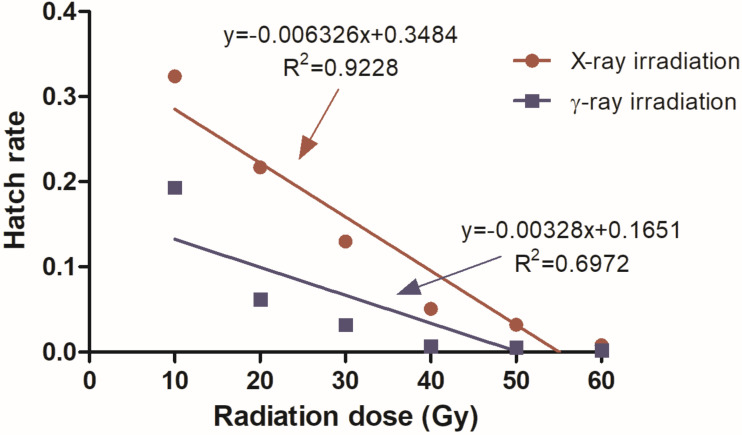
Effects of pupal irradiation on the hatch rate.

After the irradiated male pupae emerged, they mated with fertile females. Females laid eggs after the blood meal, and there was no significant difference in the number of eggs laid between them and the control group (F = 0.984, df = 12, *p* = 0.462) (Table 1). That is, any dose of γ-ray and X-ray irradiation did not affect the number of eggs laid in female mosquitoes. Nevertheless, the hatch rate of their eggs was significantly decreased with the increase in radiation dose of X-rays or γ-rays (X-rays: χ^2^ = 2717.677, df = 6, *p* < 0.001; γ-rays: χ^2^ = 3835.507, df = 6, *p* < 0.001) (Figure 1). Moreover, at the same radiation dose, the γ-ray radiation group had a lower hatch rate than the X-ray radiation group, under the same radiation dose (Table 1). However, X-ray radiation could achieve a sterility effect similar to γ-ray radiation by increasing the radiation dose. For example, after 60 Gy X-ray and 40 Gy γ-ray radiation, the hatch rates were 0.8 ± 0.6 and 0.7 ± 0.7, respectively, and the difference was not significant (Table 1).

### 2.2. Effects of Radiation on the Survival of Male Mosquitoes

**Figure 2:**
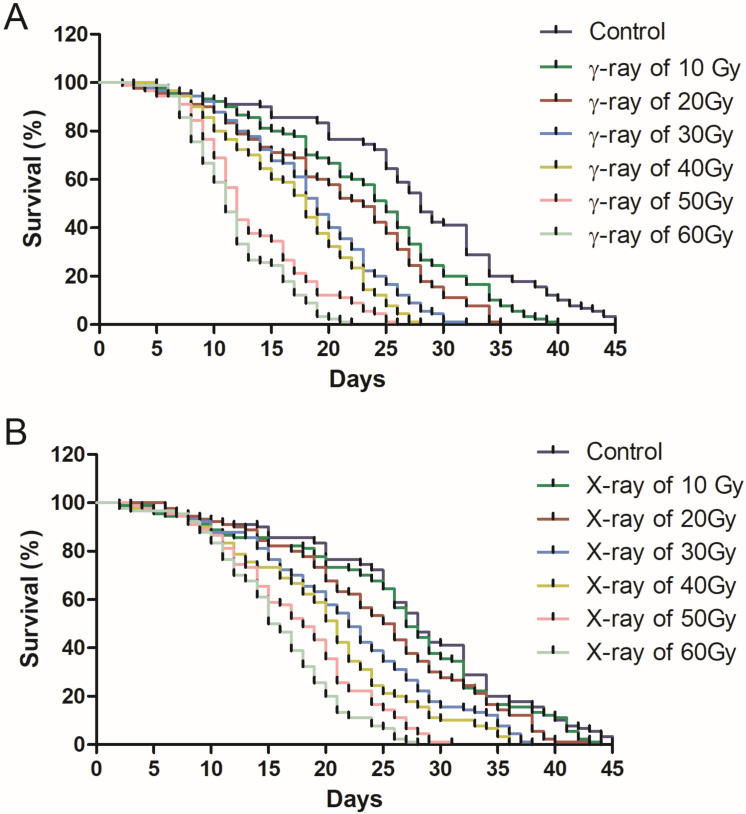
Survival curves of *Ae. albopictus* males irradiated with different doses of γ-rays (**A**) and X-rays (**B**).

Pupal radiation had a significant effect on the survival time of adult male mosquitoes after their emergence. Except for 10 Gy of X-ray irradiation, any other dose of X-ray and γ-ray irradiation significantly reduced the male survival time (Table 2). The survival time of the irradiated males was inversely proportional to the radiation dose (Figure 2). However, γ-rays significantly affected male survival time more than X-rays. At the same dose, the average survival times of X-ray-exposed males were significantly longer than those exposed to γ-rays (Table 2). Male mosquitoes exposed to a 40 Gy dose of X-ray radiation lived for 20.1 days, but males exposed to a 40 Gy dose of γ-ray radiation lived for only 17.1 days. Nevertheless, there was no significant difference in the survival time between the males under 40 Gy X-ray radiation and 20 Gy γ-ray radiation (*χ*^2^ = 0.375, df = 1, *p* = 0.54). When exposed to X-rays of 60 Gy and γ-rays of 40 Gy, the average survival time was also the same (*χ*^2^ = 2.393, df = 1, *p* = 1.22). These results suggest that γ-ray radiation significantly reduces male longevity compared with X-ray radiation at the same dose.

### 2.3. Effects of Radiation on Male Mating Competitiveness

From the above results, after 60 Gy X-ray or 40 Gy γ-ray irradiation, the male survival time exceeded 15 days, and the hatch rate was less than 1%. Thus, X-rays of 60 Gy and γ-rays of 40 Gy were chosen to test the male mating competitiveness of the irradiated males. After mating with the irradiated males, the hatch rate of fertile females decreased with the increase in release ratios, and the difference was significant (X-rays: χ^2^ = 1741.535, df = 5, *p* < 0.001; γ-rays: χ^2^ = 1882.469, df = 5, *p* < 0.001) (Table 3). Furthermore, the difference in the average hatch rate between the two types of irradiation at the same release ratios was not statistically significant.

**Figure 3:**
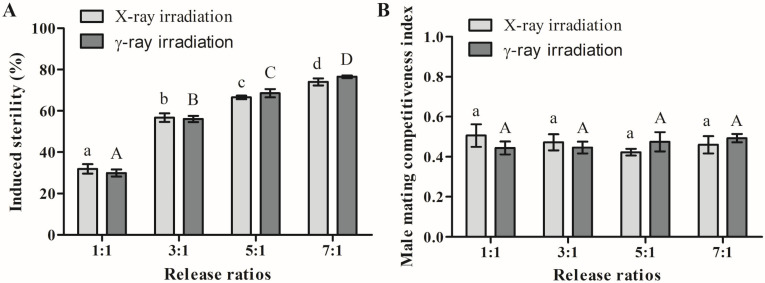
Effects of X-ray and γ-ray irradiation on the induced sterility (**A**) and male mating competitiveness index (**B**) of *Ae. albopictus.* Error bars indicate ± SE. Values followed by different letters (a–d or A–D) are significantly different from one another (ANOVA and Tukey’s post hoc tests).

In addition, the induced sterility gradually increased as the release ratio increased, with a significant difference (Figure 3A). When the release ratio exceeded 3:1, the average induced sterility by the two types of radiation exceeded 50%. When the release ratio reached 7:1, the average induced sterility after γ-ray and X-ray irradiation reached 76.5% and 74.0%, respectively. There was no significant difference between γ-ray and X-ray irradiation at the same release ratios in induced sterility.

Moreover, both γ-ray and X-ray irradiation reduced the male mating competitiveness index (*C*). Nonetheless, *C* was independent of the release ratio for the two types of radiation (Figure 3B). After γ-ray and X-ray irradiation, the average *C* was 0.46 ± 0.03 and 0.47 ± 0.04, respectively. This means that the sterile males were approximately half as competitive as the normal males.

## 3. Discussion

Emerging and re-emerging arboviral diseases, including dengue, Zika, and chikungunya, pose severe threats to human health [24]. As one of the vectors of arbovirus, *Ae. albopictus* has spread from south Asia to at least 70 countries worldwide in recent decades [25,26]. SIT helps suppress mosquito populations, thereby reducing the spread of mosquito-borne diseases [27]. However, there are still many problems to be solved [18]. For example, the sterility effects of different types of radiation and their impact on mosquito lifespan and mating competitiveness need to be addressed [28]. Although many reports have shown the sterility effects of γ-rays and X-rays on mosquitoes, there is no direct comparison of the two types of radiation on *Ae. albopictus*. In this study, the impact of γ-rays and X-rays was mainly assessed by the emergence rate, egg number, hatch rate, survival, induced sterility, and male mating competitiveness index of *Ae. albopictus*.

Based on our results, only high doses of γ-rays, such as 50 and 60 Gy, reduced the emergence rate (Table 1). In contrast, the hatch rate was significantly reduced in each irradiation group and showed a dose-dependent relationship. Radiation causes overt lethal mutations that lead to embryonic death after fertilization [29]. Similarly, Bond et al. irradiated *Ae. albopictus* with γ-rays of 40 Gy, and the fertility dropped to 0.87% [30]. In addition, the hatch rate of male pupae over 12–36 h irradiated using X-rays by Du et al. was similar to our results [19]. Balestrino et al. showed that male *Ae. albopictus* pupae can achieve 99% sterility when exposed to γ-rays of 40 Gy, which is consistent with our results [31].

Mosquito somatic cells can be damaged by radiation, thereby reducing the survival time of radiated males [20]. The radiation of pupae of different ages may cause different degrees of damage. In this study, 12–24 h old male pupae were exposed to X-rays and γ-rays. The survival time decreased with increasing radiation doses of X-rays and γ-rays. At the same dose, γ-rays were more harmful to mosquitoes than X-rays, but they were better at inducing sterility. Furthermore, we found that males irradiated using γ-rays of 20 Gy or X-rays of 40 Gy were not significantly different in terms of emergence rate, hatch rate, or average survival time. The differences in emergence rate, hatch rate, and survival time after γ-rays of 40 Gy and X-rays of 60 Gy were also not statistically significant (Table 1 and Table 2). Yamada et al. irradiated pupae with the same dose of X-rays; adult mosquitoes generally had lower lifespans than those in our experiments, and the hatch rate was lower [20]. Possible reasons are the different sources of the mosquitoes, and the fact that sterile males and fertile females in our experiment were released in a 1:1 ratio, while in Yamada et al. they were released in a 6:5 ratio. Based on the above results of our experiments, we can achieve the same sterility effect as low-dose γ-rays by increasing the radiation dose of X-rays. In conclusion, we could replace γ-rays with high-dose X-rays, but the impact of field release after replacement still needs to be further studied.

Therefore, we irradiated males with γ-rays of 40 Gy and X-rays of 60 Gy and then simulated field experiments. Sterile male mating competitiveness and induced sterility were measured at different release ratios (sterile males/fertile males). We found that when the male release ratio was higher, the rate of induced sterility was higher, and the hatch rate was lower (Figure 3A and Table 3). At a release ratio of 7:1, the egg hatching rates of γ-ray and X-ray irradiations were 20.6 ± 1.6% and 22.1 ± 4.3%, respectively, and the induced sterility of both types of radiation was close to 80%. Similarly, Du et al. reported that after X-ray irradiation, the induced sterility of *Ae. albopictus* was 74.1%, and the hatching rate was 21.1% [19]. Bond et al. irradiated male pupae with γ-rays of 40 Gy, and the egg hatch rates achieved at release ratios of 1:1 and 5:1 of sterile and fertile males were 54.3% and 22.6%, respectively [32]. In this study, regardless of the proportion released, the male mating competitiveness index was maintained between 0.4 and 0.6, which is not high (Figure 3B).

Interestingly, a study by Madakacherry et al. showed that increasing the release ratio of sterile males could eliminate the effect of weakened male competitiveness [33]. In addition, Bellini et al. showed that in semi-field enclosure experiments, radiation caused premature maturation of sterile males, which led to the fact that sterile males could mate with females earlier than fertile males [34]. In this study, at the same proportion of sterile males to untreated males, there were no statistically significant differences in the high-dose X-ray and low-dose γ-ray hatching rates, induced sterility, or male mating competitiveness index. Thus, high-dose X-rays could replace low-dose γ-rays for the sterile treatment of *Ae. albopictus*, especially when γ-rays are not readily available or are expensive.

Nevertheless, one of the reasons why SIT is not widely used in mosquito control is that it is difficult to irradiate males without reducing male mating competitiveness and survival [35]. Rodriguez et al. found that ethanol, trimethylglycine, and beer could be used as radiation protection agents to extend the survival time of irradiated males [36]. In recent studies, a combination of incompatible and sterile insect technology produced large-scale male sterility with a low irradiation dose without affecting male mating competitiveness and survival [35,37]. In addition, Becker et al. proved that a combination of *Bacillus thuringiensis israelensis* and SIT is effective for *Ae. albopictus* control, which has no negative impact on public health or the environment [17].

Moreover, some novel SIT methods have been developed and applied to mosquito control. For instance, a novel SIT was recently reported, which first treated larvae with dsRNA and then exposed pupae to thiotepa to make the mosquitoes sterile [38]. This novel SIT reduced the mosquito population in the field, reducing the dengue fever incidence rate [38]. Furthermore, the same species of mosquitoes from different geographical locations have similar susceptibilities to irradiation, indicating that it is possible to produce a sterile male mosquito for the cross-border application of SIT to reduce costs [39,40]. Overall, SIT is expected to be more effective and low-cost in suppressing mosquito populations in the future.

In conclusion, this study mainly compared the sterility effect on *Ae. albopictus* of SIT using X-rays or γ-rays. We suggest that high-dose X-rays can be used instead of γ-rays when sterile males are released into the field, and that the higher the release ratio, the more significant the population suppression. In general, our experiments provide a feasible idea for reducing the cost of releasing many sterile males into the field, which is substantial for mosquito control.

## 4. Materials and Methods

### 4.1. Mosquito

*Ae. albopictus* mosquitoes from Foshan City, Guangdong Province, China, were collected by the Guangdong Provincial Center for Disease Control and Prevention. This colony was maintained in an insectary under climate-controlled conditions at a temperature of 28 ± 1 °C, relative humidity of 80 ± 5%, and a photoperiodic regime of 14 L:10 D. The mosquito larvae were fed turtle food, and the adults were provided with a 10% glucose solution ad libitum. Adult females were fed with Kunming mouse blood (provided by the Animal Experiment Center of Anhui Medical University) to lay eggs.

### 4.2. Radiation Effects on Emergence Rate, Egg Number, Hatch Rate, and Survival

The Biobeam GM2000 γRay Irradiation device (Gamma-Service Medical GmbH, Leipzig, Germany) (cobalt-60 source) and Varian Clinac 23EX linear accelerator (Varian, Palo Alto, CA, USA) were used for γ-ray and X-ray radiation, respectively.

Male pupae (12–24 h old) of *Ae. albopictus* (150–200 pupae/tray) were placed in the center of the tray, and then the tray was placed in the radiation area of the γ-ray or X-ray irradiator for irradiation. The pupae were irradiated at 10, 20, 30, 40, 50, and 60 Gy at a radiation dose rate of 200 Gy/h. Subsequently, the pupae were moved to new mosquito cages (25 cm × 35 cm × 25 cm). Those pupae without ordinary emergence within 48 h were recorded to calculate the emergence rate. The control group was the same as the radiation group. The exact number of female virgin mosquitoes was added to the cage for mating. Blood feeding was carried out 2 days later. The engorged mosquitoes were placed individually into a 9-ounce cup containing wet funnel-shaped filter paper for oviposition. The eggs were collected and counted 5 days after blood feeding. Then, the mosquito eggs were dried for 24 h and placed in a water basin for 7 days for hatching. The egg number and hatch rate were used to evaluate female fertility. Moreover, pupae exposed to 10, 20, 30, 40, 50, and 60 Gy were used to assess the effects of γ-ray or X-ray radiation on the survival of male adults in the F1 generation. The survival time of each mosquito was recorded until the last mosquito died. Three replicates of 30 male pupae or adults were used in each group.

### 4.3. Radiation Effects on Male Mating Competitiveness

Based on the results of the first experiment (the treatment that minimized the effect on survival with maximum sterility), pupae exposed to γ-rays of 40 Gy and X-rays of 60 Gy were used to assess the impact of irradiation on the male mating competitiveness of *Ae. albopictus*.

Male pupae (12–24 h old) were irradiated with γ-rays of 40 Gy or X-rays of 60 Gy and then moved to new mosquito cages (25 cm × 35 cm × 25 cm) for emergence. Then, 30, 30, 90, 150, 210, and 0 treated males were transferred to separate mosquito cages comprising 0, 30, 30, 30, 30, and 30 fertile males. The release ratios (sterile/fertile; S/F) were 1:0, 1:1, 3:1, 5:1, 7:1, and 0:1. After 1 day, 30 fertile female virgin mosquitoes (5–7 days old) were transferred to each mosquito cage. Three days later, the mosquitoes were fed blood. Each blood-fed female was transferred to a new cup for oviposition. The number of eggs laid by each mosquito was recorded, and the eggs were permitted to hatch for 7 days.

The hatch rate of each group was recorded. The experiment was repeated three times in each group. The following formulae were used to calculate induced sterility (IS) [41] and the male mating competitiveness index (*C*) [42]:

$$\mathrm{IS}=\left(1-\frac{H_{1}}{H_{2}}\right)\times 100\%$$

$$C=\frac{H_{2}-H_{1}}{H_{1}-H_{0}}\times \frac{N_{1}}{N_{2}}$$

where H_0_ is the hatch rate of the irradiated control group, H_1_ is the hatch rate of the competition group (involving 3:1, 5:1, and 7:1 sterile to fertile males), H_2_ is the hatch rate of the fertile control group, N_1_ is the number of fertile males, and N_2_ is the number of irradiated males.

### 4.4. Statistical Analysis

All statistical analyses were performed with IBM SPSS version 20. Pearson’s chi-square and Bonferroni tests were used to compare differences between each group’s emergence and hatch rates. Differences in the egg number, IS, and *C* between groups were compared using ANOVA and Tukey’s post hoc tests. Kaplan–Meier analysis and log-rank (Mantel–Cox) tests were used to determine the difference in survival among the groups. All data are presented as the mean ± standard error (SE). A value of *p* < 0.05 was considered statistically significant.

## Figures and Tables

**Table 1:** Effects of pupal irradiation on emergence, egg number, and hatch rate.

Irradiation	Emergence Rate (%)	Egg Number	Hatch Rate (%)
Control	94.1 ± 1.4 ^a^	61.7 ± 1.8 ^a^	86.1 ± 4.4 ^a^
X-ray of 10 Gy	93.7 ± 2.5 ^a^	62.4 ± 2.3 ^a^	32.4 ± 3.7 ^b^
X-ray of 20 Gy	92.6 ± 2.1 ^a^	67.0 ± 2.3 ^a^	21.7 ± 2.1 ^c^
X-ray of 30 Gy	91.9 ± 2.8 ^a^	63.6 ± 2.2 ^a^	13.0 ± 1.6 ^d^
X-ray of 40 Gy	91.5 ± 4.2 ^a^	62.1 ± 2.2 ^a^	5.1 ± 1.8 ^e^
X-ray of 50 Gy	91.9 ± 3.1 ^a^	62.9 ± 2.0 ^a^	3.2 ± 1.0 ^e^
X-ray of 60 Gy	89.6 ± 4.3 ^a^	64.6 ± 2.1 ^a^	0.8 ± 0.6 ^f^
γ-ray of 10 Gy	91.9 ± 2.8 ^a^	65.1 ± 2.1 ^a^	19.3 ± 2.2 ^c^
γ-ray of 20 Gy	91.1 ± 2.2 ^a^	59.3 ± 2.1 ^a^	6.2 ± 1.2 ^e^
γ-ray of 30 Gy	87.4 ± 2.1 ^a,b^	61.0 ± 2.2 ^a^	3.2 ± 0.9 ^e^
γ-ray of 40 Gy	86.7 ± 2.7 ^a,b,c^	64.7 ± 2.3 ^a^	0.7 ± 0.7 ^f^
γ-ray of 50 Gy	78.9 ± 3.1 ^b,c^	61.6 ± 2.2 ^a^	0.6 ± 0.5 ^f^
γ-ray of 60 Gy	75.6 ± 3.1 ^c^	62.8 ± 2.0 ^a^	0.2 ± 0.4 ^f^

Data are presented as the mean ± SE. Values followed by different letters (^a–f^) are significantly different from one another (emergence rate and hatch rate: Pearson chi-square and Bonferroni tests; egg number: ANOVA and Tukey’s post hoc tests; *p* < 0.05).

**Table 2:** Effects of pupal irradiation on the survival of male mosquitoes.

Radiation Dose (Gy)	Average Survival Time (X-rays, Days)	Average Survival Time (γ-rays, Days)
Control	27.7 ± 1.0 ^a^	
10	26.4 ± 1.1 ^a,b^	23.5 ± 0.9 ^c,d^
20	24.9 ± 1.0 ^b,c^	21.1 ± 0.9 ^f^
30	22.1 ± 0.9 ^d,e,f^	18.6 ± 0.7 ^g^
40	20.1 ± 0.8 ^e,f^	17.1 ± 0.6 ^h,i^
50	17.7 ± 0.7 ^g,h^	13.3 ± 0.6 ^j^
60	15.9 ± 0.6 ^i^	11.8 ± 0.4 ^k^

Data are presented as the mean ± SE. Values followed by different letters (^a–k^) are significantly different from each other (Kaplan–Meier analysis and log-rank (Mantel–Cox) test, *p* < 0.05).

**Table 3:** Egg hatch rates at different release ratios.

Release Ratio (S/F)	Hatch Rate (γ-rays, %)	Hatch Rate (X-rays, %)
0:1	89.2 ± 3.8 ^a^	87.4 ± 2.4 ^a^
1:1	62.1 ± 4.1 ^b^	59.0 ± 6.6 ^b^
3:1	38.8 ± 3.3 ^c^	37.2 ± 4.9 ^c^
5:1	27.6 ± 4.0 ^d^	28.7 ± 1.9 ^d^
7:1	20.6 ± 1.6 ^e^	22.1 ± 4.3 ^e^
1:0	0.4 ± 0.5 ^f^	0.7 ± 0.7 ^f^

S/F means sterile/fertile. Data are presented as the mean ± SE. Values followed by different letters (^a–f^) are significantly different from one another (Pearson chi-square and Bonferroni tests, *p* < 0.05).

## Data Availability

Available on demand.
